# Abdominal compartment syndrome caused by tension pneumoperitoneum in a scuba diver

**DOI:** 10.1308/003588412X13373405385773

**Published:** 2012-05

**Authors:** J Bunni, PJ Bryson, SM Higgs

**Affiliations:** ^1^North Bristol NHS Trust,UK; ^2^Diving Diseases Research Centre, Plymouth,UK

**Keywords:** Tension pneumoperitoneum, Abdominal compartment syndrome, Scuba diving, Barotrauma

## Abstract

Abdominal compartment syndrome is a surgical emergency caused by a raised intra-abdominal pressure, which may lead to respiratory, cardiovascular and renal compromise. It is most commonly seen in post-operative and trauma patients and it has a variety of causes. Tension pneumoperitoneum (TP) is a rare cause of abdominal compartment syndrome most often seen after gastrointestinal endoscopy with perforation.

We present the case of a fit 52-year-old experienced female diver who developed TP and shock following a routine training dive to 27m. Following accidental inhalation of water, she had an unstaged ascent and, on reaching the surface, developed severe acute abdominal pain and distension. She was brought to our emergency department by air ambulance for assessment. Clinical and radiological examination revealed a shocked patient with dramatic free intra-abdominal gas and signs of abdominal compartment syndrome, which was treated with needle decompression. Symptoms and signs resolved quickly with no need for further surgical intervention. TP is a surgical emergency where surgery can be avoided with prompt diagnosis and treatment.

Tension pneumoperitoneum (TP) is a variant of abdominal compartment syndrome (ACS) with high intra-abdominal pressure caused by trapped gas leading to decreased venous return, decreased visceral perfusion and splinting of the diaphragm with consequent circulatory and respiratory compromise. It is an uncommon condition that most commonly results from gastrointestinal perforation during upper and lower gastrointestinal endoscopy with extravasation of insufflating gas. It has also been described after perforation proximal to an obstructing lesion in the gut, following blunt abdominal trauma, mechanical ventilation, pulmonary blast injuries and tracheal injury as well as following nasopharyngeal oxygen delivery and mouth-to-nose resuscitation in an infant.^1,2^

Gastric barotrauma occurs rarely during diving accidents due to swallowed air and an uncontrolled ascent from depth.^3^ Trapped air in the stomach expands rapidly as the pressure decreases and ruptures the stomach, often along the lesser curve. This usually (but not always) requires surgery for peritoneal lavage and gastric repair.^4^ We present a case of barotrauma leading to a TP and ACS in a scuba diver.

## Case history

A previously fit and well 52-year-old woman was brought to hospital with acute severe abdominal pain. She was an experienced diver undergoing a routine training dive in a quarry lake with an instructor. She had an uneventful dive down to 27m, using a trimix breathing gas. This contained a mixture of oxygen (32%), helium (10%) and nitrogen (58%). She was wearing a dry suit and an under suit below. During the exercise at 27m, she accidentally replaced her mouthpiece or regulator upside down, which resulted in inhalation of a small amount of water and air swallowing. She then had a rapid but controlled ascent without staged stops and developed acute severe abdominal pain on reaching the surface.

The patient was brought to hospital by air ambulance and primary survey revealed an intact airway, hypoxia with oxygen saturations of 91% on room air and a respiratory rate of 20 breaths per minute. Her systolic blood pressure was 70mmHg and the pulse rate was 90 beats per minute. Abdominal examination revealed a grossly distended peritonitic abdomen.

**Figure 1:**
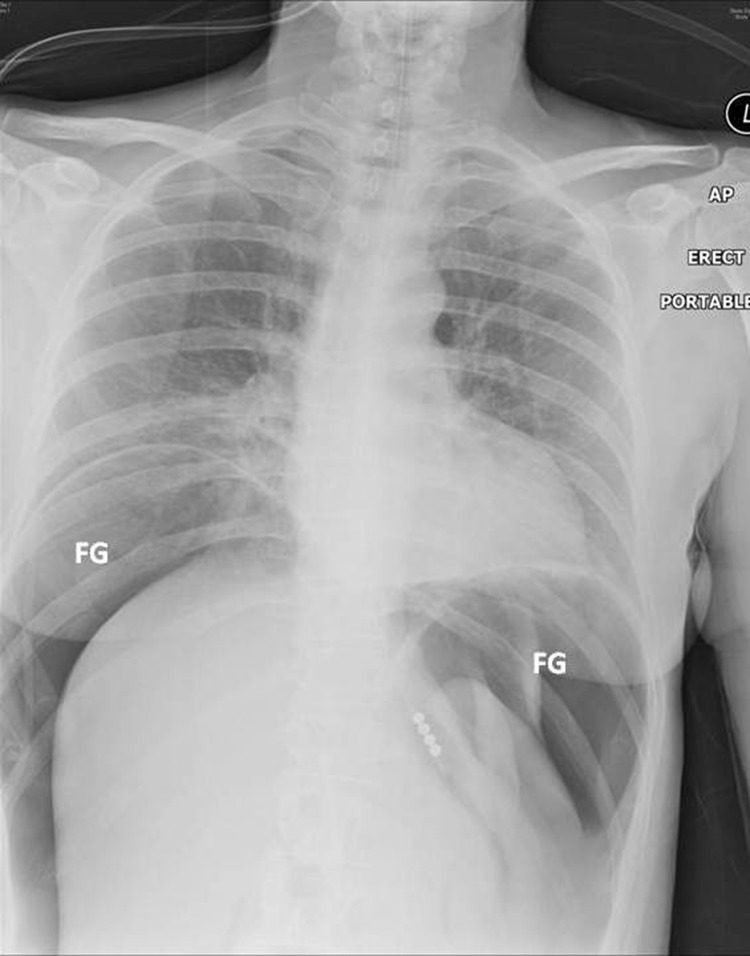
Chest x-ray showing free air under the diaphragm but no evidence of pneumomediastinum

**Figure 2:**
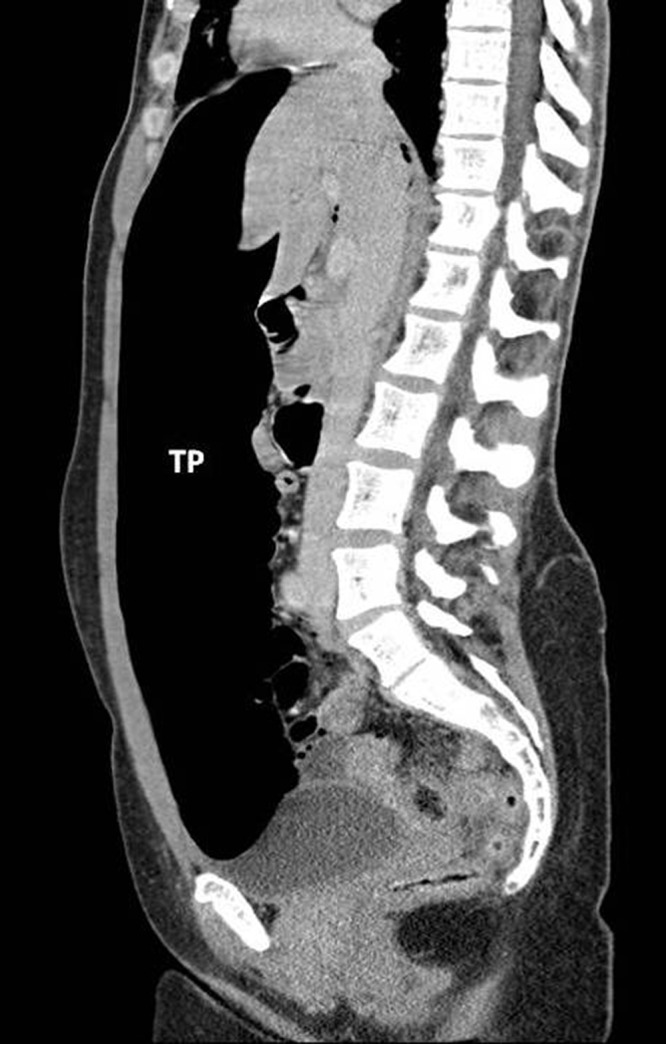
Computed tomography with oral contrast showing pneumoperitoneum with compression of intra-abdominal viscera

**Figure 3:**
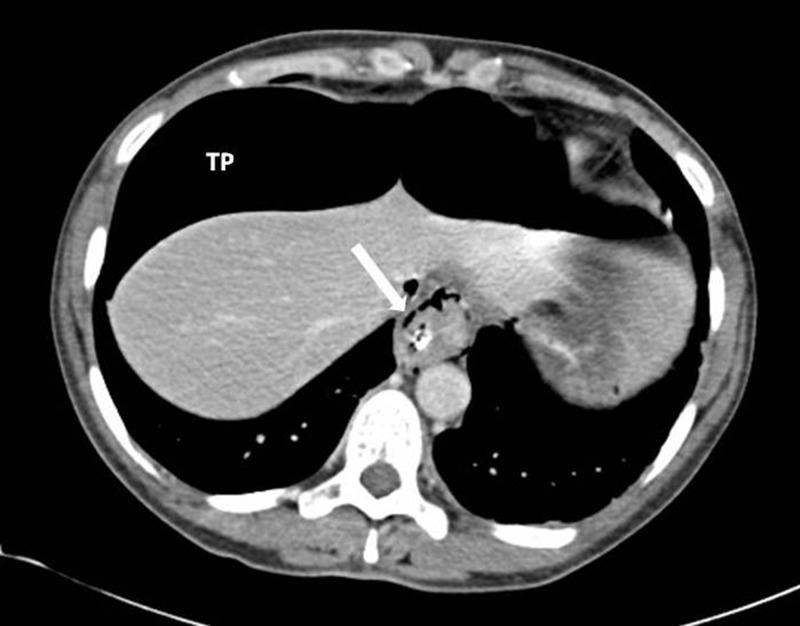
Computed tomography showing a small amount of gas in the soft tissues

The differential diagnosis was gastric barotrauma due to air swallowing resulting in free perforation and peritonitis. Following intravenous fluid administration and placement of a Ryle’s nasogastric tube, an erect chest x-ray was performed that revealed a large amount of free air under the diaphragm but no evidence of pneumomediastinum (Fig 1). Urgent computed tomography (CT) with oral contrast showed massive pneumoperitoneum with compression of intra-abdominal viscera (Fig 2). There was no evidence of pneumothorax but in the lower mediastinum there was a small amount of gas in the soft tissues (Fig 3).

Needle decompression using a 14G cannula percutaneously through the abdominal wall in the left iliac fossa was performed as a temporising procedure prior to an exploratory laparotomy. There was an audible release of gas under pressure and a dramatic improvement in the patient’s physiological parameters with immediate resolution of pain and clinical signs.

Given the rapid improvement and lack of any evidence of contrast leakage on CT, surgery was postponed and the patient was admitted for overnight observation. She was monitored closely for symptoms and signs of decompression illness after discussion with the local Diving Diseases Research Centre. She remained stable with no further symptoms. The volume of nasogastric tube drainage was zero and the tube was subsequently removed. There was no residual abdominal tenderness, blood tests were all normal and there was no pain on drinking clear fluids or eating. She was discharged home the next day and remained well at follow-up.

## Discussion

ACS is a serious illness characterised by organ dysfunction secondary to intra-abdominal hypertension. The aetiology of ACS can be split into three categories depending on the cause:

> *Primary ACS*: Intra-abdominal pathology is directly responsible for ACS, eg intraperitoneal haemorrhage from penetrating trauma or a ruptured abdominal aortic aneurysm or pancreatitis.
> *Secondary ACS*: Extra-abdominal injury leads to intra-abdominal fluid collection, eg fluid overload and burns.
> *Chronic ACS*: This is secondary to cirrhosis with ascites, morbid obesity and peritoneal dialysis.

The aim of management should be prompt diagnosis and to maintain an adequate abdominal perfusion pressure. This can be achieved using a variety of techniques. The intra-abdominal pressure can be reduced by increasing abdominal wall compliance using analgesia, sedation and neuromuscular blockade where appropriate. Intraluminal and extraluminal pressure should be lessened by the placement of a nasogastric tube and urinary catheter and by drainage of compressing fluids where possible by abdominal paracentesis. Mean arterial perfusion should be optimised by cautious fluid replacement and the use of vasopressors if necessary. In a patient with primary ACS, a laparotomy and treatment of the cause may be necessary and should not be delayed. Temporary abdominal closure is essential in some cases if primary closure is impossible or a second look laparotomy is likely to be required.

Rapid ascent by divers from depth causes a rapid increase in volume of trapped gas in hollow organs as the environmental pressure decreases (decompression). Decompression illness is a distinct entity and is caused by bubbles in blood or tissue during or after decompression. It includes two pathophysiological syndromes: arterial gas embolism and the more common decompression sickness. Arterial gas embolism occurs when expanding gas ruptures alveolar capillaries, allowing gas to enter the arterial circulation (pulmonary barotrauma). Decompression sickness is caused by the formation of bubbles of dissolved gas (usually nitrogen) in tissues. The bubbles can manifest as mechanical, embolic and biochemical phenomena ranging from minor to fatal.^5^

The exact mechanism of our patient’s TP was not clearly identified. Possible sources of entry were from pulmonary or gastric barotrauma. Breath-holding during ascent from a dive is well recognised to cause pulmonary barotrauma and subsequent passage of air from lung to abdominal cavity via the mediastinum has been described in a diver.^4^ Although there was a small amount of pneumomediastinum in this case, there was no pneumothorax as one might expect with such a dramatic amount of gas. The small bubbles of gas seen in the mediastinum may have been present due to the extremely high intra-abdominal pressure.

Air swallowing during an unstaged ascent during diving can cause rapid expansion of gas and subsequent rupture of the stomach, usually along the lesser curve. TP has been reported in a diver ascending rapidly from a depth of 65m who exhibited other features of decompression illness and who required an emergency laparotomy and hyperbaric oxygen therapy.^6^ In a small series of patients with gastric perforation complicating diving accidents, surgery was performed in the majority.^7^ There were no signs of gastric perforation found at laparotomy in that case despite careful examination of the gastrointestinal tract. Our case appears to be similar in nature but was managed in a markedly different manner. Prompt decompression of the pneumoperitoneum allowed a measured assessment of the patient and it was clear after a period of observation that no further intervention was necessary.

## Conclusions

ACS is a relatively common condition but the causes are not always clear. Barotrauma from diving accidents does not usually present to the general surgeon on call and TP should be considered in this patient group presenting with abdominal pain and distension. Prompt diagnosis and management may make immediate surgery unnecessary but close observation and repeat examination are essential to avoid delaying surgery when indicated. Symptoms and signs of decompression illness should also be looked for and, if suspected, should prompt further discussions with a recompression facility.
